# Characterization of Hot-Applied Joint Sealants and Their Components in Terms of Their Chemical Composition and Basic Performance Properties

**DOI:** 10.3390/ma16196490

**Published:** 2023-09-29

**Authors:** Justyna Stępień, Eva Remišová

**Affiliations:** 1 Department of Transportation Engineering, Faculty of Civil Engineering and Architecture, Kielce University of Technology, Aleja Tysiąclecia Państwa Polskiego 7, 25-314 Kielce, Poland; 2 Department of Highway and Environmental Engineering, Faculty of Civil Engineering, University of Žilina, Univerzitná 8215/1, 010 26 Žilina, Slovakia; eva.remisova@uniza.sk

**Keywords:** hot-applied joint sealant, FTIR, EDX, asphalt binder, mineral filler, rubber crumb

## Abstract

Hot- and cold-applied joint sealants are materials commonly used for the surface repairs of the upper layers of asphalt or concrete road surfaces. Our investigations covered six hot-applied joint sealants, classified as the high-extension type N1 (elastic) or low-extension type N2 (normal), in accordance with the standard EN 14188-1; the sealants were obtained commercially from four European manufacturers. The present paper focuses on the characterization of the consistency of the joint sealants, the bituminous binders that contain them, and the characterization of their insoluble components. Additional testing methods included an FTIR analysis of the sealants and the extracted binders, as well as SEM, EDS, and sieving analyses of the insoluble material. Joint sealants are complex formulations and include a broad range of base asphalt binders and other components. Their compositions may vary widely, while still fulfilling the performance specifications. Through the extraction of the solvents and the separation of the crack sealants, it was found that radically different compositions of crack sealants resulted in the comparatively similar performance of the tested material. The EDS and FTIR analysis methods provided insights into the composition of the crack sealants and the types of mineral materials used.

## 1. Introduction

Maintaining the integrity of the upper layers of road structures ensures their longer life, as well as travel comfort and safety. Preventing the degradation of road construction requires the immediate and permanent maintenance of discontinuities, which occur during pavement service [1]. Hot- and cold-applied joint sealants are materials commonly used for the surface repairs of the upper layers of road surfaces made of either asphalt mixtures or Portland cement concrete. They are used to seal cracks in the surface and to supplement expansion joints, as well as to fix small distresses. Their properties must be adapted to the nature of the job, the location where they will be used in the surface structure, and the effects of external factors [2,3,4]. The subject of the research in this article concerns a selection of the properties of hot-applied joint sealants, which are a type of thermoplastic material that requires heating and melting before application. The discontinuities in pavements are sealed by the adherence of the joint sealant to the appropriate surfaces. Subsequently, the pavement structure is protected from the infiltration of, e.g., rainwater and chemicals, and the further degradation of the surface.

The selection of the joint sealant type, in terms of its viscoelastic properties and resistance to fuel, as well as the method of application, should result from the role and function that it will perform during the service life of the road surface. As construction products, they must meet the requirements of the applicable standards. In Poland, until March 2005, the composition of joint sealants was strictly regulated, and only products with a defined composition in accordance with the industry standard BN-74/6771-04 were permitted. This composition could include a 45–50% content of D100 road bitumen, a 50-55% content of lime filler, and a 0–5% content of mineral wool. Additionally, the use of additives, such as synthetic resins, to refine the properties of the asphalt binder was permitted. In 2004, the European Committee for Standardization, CEN, introduced new standards for hot-applied joint sealants—EN 14188-1 [5]. The adopted standard did not introduce material restrictions, but it did provide a specific set of requirements that should be met by the final product depending on the function that it is expected to fulfill. Hot-applied joint sealants are divided into the following types:

- Flexible (high-extension)—type N1;
- Normal (low-extension)—type N2;
- High-extension and fuel-resistant—type F1;
- Low-extension and fuel-resistant—type F2.

Countries such as Canada and the United States use the ASTM [6] standards for the quality control of crack and joint sealants [4], resulting in a different classification to that used in European countries. This document specifies the maximum flow and cone penetration specifications for each of the four types (I-IV) of crack sealants. Based on their service environment (moderate, mostly cold, and very cold climates), most types are selected based on the lowest temperature they could encounter while in service.

In pavement engineering, various technological solutions are used in the field of asphalt modification, and the use of additives for mineral and asphalt mixtures depends on their purpose in the road surface construction, the environmental conditions, and the pro-ecological requirements [7,8,9,10]. The use of neat asphalt binders in crack sealants provides a cheap solution for the maintenance of cracking pavements; however, such formulations are prone to debonding and poor low- or high-temperature performance, depending on the grade of the base asphalt binder. Therefore, modified asphalt binders are used [11,12,13].

One of the basic parameters for joint sealants that must be ensured is good adhesion to various types of surfaces [4]. Cao E. et al. [2] investigated three environmental factors, namely temperature, water, and contamination, to determine the effects of adverse environmental exposures on the various crack sealants commonly used in hot and moderate climates; they used a tensile test to illustrate adhesion failure between the sealant and the crack wall.

Depending on the requirements and purpose, it is possible to regulate the properties of a joint sealant by changing its chemical composition and the proportion of its components—the binders, fillers, additives, and modifiers of the binders [11,14,15,16]. Styrene–butadiene–styrene (SBS) and rubber crumb CR (10, 15, 20%) have been utilized to modify asphalt-based sealants to overcome the disadvantages of the poor high-temperature and rheological properties of sealants [17]. The SBS/CR-modified asphalt sealant has a greater viscosity and higher temperature deformation resistance.

The results of research on the effects of different fillers on the heat resistance, elasticity, flexibility, and performance of polymer-modified mastics at low temperatures have been highlighted by Gnatenko et al. [18]. To reduce brittleness and increase the flexibility of joint sealants and mastics at low temperatures, it was necessary to use a plasticizer. On the other hand, improving their performances at high temperatures required the use of thermoplastic elastomers made of SBS types, latexes, mineral fillers, or fine crumb rubbers, which are similar to those used in road paving asphalt binders [19,20]. The content levels of the mineral fillers in the investigated sealants were 5, 10, and 15%, while those of the crumb rubber were considered to be 3, 5, 15, and 20%. It has been established that an increase in the content of mineral fillers in a sealant’s composition results in a 15% increase in the softening point (by as much as 4–8 °C); however, it significantly decreases its flexibility at low temperatures. The flexibility at low temperatures for a polymer bitumen sealant was preserved down to a temperature of less than −35 °C. It was found that the best results were achieved when crumb rubber was used as the filler, resulting in the highest softening temperature increase and the least detrimental effects to the low-temperature properties of the mixture, as compared to mineral fillers. It was also found that the combination of plasticized bitumen and an SBS-type polymer and a cationic polymer latex allows for the obtainment of a sealant characterized by both high elasticity and flexibility at low temperatures and heat resistance.

Michta et al. conducted investigations [21] on the optimal composition of joint sealants with highly modified asphalt. The sealants included mineral or mixed filler used in amounts equaling 20, 40, and 60%. In its composition, the mixed filler contained hydrated lime (calcium hydroxide) in amounts equaling 10, 20, and 30%, used interchangeably with lime filler. The best parameters (needle penetration and softening point) characterized the joint sealant with a content of 80% highly modified asphalt and 20% mineral filler containing 20% hydrated lime. The beneficial effect of the addition of rapeseed oil in an amount equaling 3% on the lowering of the Fraass breaking point was also established. Hydrated lime used in these investigated sealants may act as an active filler that has antioxidant and other effects, as other studies show [22,23,24]. In [22], the chemical approach was evaluated with Fourier transform infrared (FTIR) spectroscopy.

As shown above, joint sealants are typically complex formulations and, given the broad range of base asphalt binders, modifiers, fillers, and other non-soluble additives, their composition may vary widely while still fulfilling the performance specifications. In this study, a forensic-like investigation was carried out on different hot-applied joint sealants to characterize their basic properties and composition.

The conducted study showed that the investigated joint sealants were characterized by highly varying contents of soluble asphalt binder, mineral filler, and other components, e.g., crumb rubber. It was found that distinctly different compositions of the crack sealants could result in comparatively similar performances of the tested material given different characteristics of the constituent materials. The use of EDX spectroscopy and Fourier transform infrared spectroscopy (FTIR) methods allowed for the identification of the chemical composition of joint sealants and their individual soluble and non-soluble components.

This work utilized physical testing, component chemistry, and functional group analysis to study the sealants. The investigation utilized an expanded scope of tests and identification methods to analyze the joint sealants. The solvent extraction method was applied to study the properties of both the joint sealants and the bituminous binders that contain them, and the EDS and FTIR methods were used to characterize their components, allowing for the comparison of different formulations in terms of their compositions. Such a combined use of different identification methods is not often seen in the evaluation of joint sealant materials. The presented results may provide a basis for future research on the performances of different joint sealant formulations. Investigations into different commercially available joint sealants may also serve a purpose in obtaining economically and ecologically favorable solutions in this regard, e.g., the utilization of waste rubber materials and fine aggregates.

## 2. Materials and Methods

### 2.1. Materials

The investigations considered hot-applied joint sealants that were classified in accordance with the standard EN 14188-1 [5] as the elastic, high-extension N1 type (marked with the symbols JS1, JS3, JS5) or the normal N2 type (marked as JS2, JS4, and JS6). They were obtained from four different manufacturers.

According to the technical specifications of the selected products, they are designed for filling expansion joints and joints in all kinds of road surfaces. Table 1 presents the characteristics of the joint sealants according to EN 14188-1 [5] and to the declarations made by manufacturers regarding the tested sealants.

**Figure 1:**
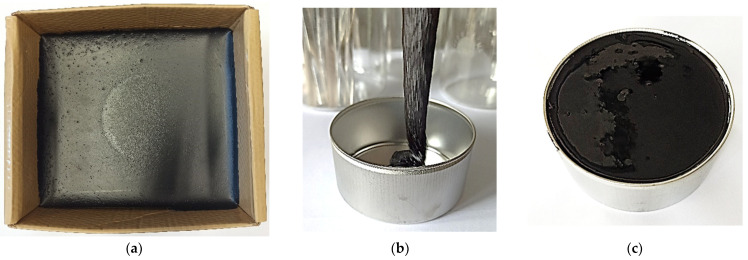
Photographs of selected investigated joint sealant: (**a**) in the original manufacturer’s packaging; (**b**) after heating up to temp. 170 °C; (**c**) in laboratory utensil.

Figure 1 shows photographs of a selected investigated joint sealant in the original manufacturer’s packaging and in a laboratory utensil after the sealant was divided into test samples.

Some of the characteristics of the joint sealants required in [5], such as the density and softening point for the JS5 sealant and the bonding strength for JS2, as well as the cohesion for JS2 and JS4, were not specified in the manufacturers’ declarations regarding the performance of the product.

The manufacturers of the JS1 and JS3 sealants have indicated different bonding strengths and cohesion test methods to those indicated in the standard for N1-type sealants; thus, only the requirements for N2 sealants for higher test temperatures were used.

The manufacturer of sealant JS6 declared compliance with the requirements of the EN 12,593 standard with regard to the Fraass breaking point by providing a value of ≤−30 °C. This parameter is not required according to EN 14188-1, but it is still valuable for the assessment of the elasticity of the tested material at low temperature.

### 2.2. Methods

#### 2.2.1. Testing Methods for Joint Sealant and Soluble Asphalt Binder

The investigation focused on the consistency characteristics of the joint sealants and the properties of their constituents and did not evaluate the properties related to the adhesion of sealants to various types of surfaces; such an evaluation requires a separate group of tests using primers.

The testing methodologies included:

- The solvent extraction of the investigated joint sealant with the use of tetrachloroethylene as a solvent:
  ◦ The determination of the soluble binder content (EN 12697-1);
  ◦ The recovery of soluble bitumen with the use of a rotary evaporator (EN 12697-3+A1).
- Basic properties of the joint sealant and soluble asphalt binder:
  ◦ Needle penetration at 25 °C (EN 1426);
  ◦ Cone penetration at 25 °C (EN 1426, EN 1388-2)—only joint sealants;
  ◦ Softening point, ring, and ball (EN 1427);
  ◦ Fraass breaking point (EN 12593);
  ◦ Elastic recovery at 25 °C (EN 13398).
- Fourier transform infrared spectroscopy (FTIR) using the attenuated total reflectance (ATR-FTIR) method.

The vacuum evaporator distillation procedure suitable for the solvent was used in accordance with EN 12697-3+A1 (Table 2).

The Thermo-Scientific Nicolet iS 5 FTIR Spectrometer and the PIKE Technologies GladiATR attenuated total reflectance accessory with a diamond window was used for the Fourier transform infrared analysis. The FEI Quanta Feg 250 SEM scanning electron microscope was used for elemental analysis.

#### 2.2.2. Testing Methods for Non-Soluble Components of Joint Sealants

For the non-soluble components of the joint sealants, the testing methodology included:

- Sieving analysis of non-soluble components:
  ◦ Mineral filler grading using the standard 0.063 mm sieve (EN-933-1)—mass of fines passing through the 0.063 mm sieve;
  ◦ Mineral and rubber dust using a standard sieve 0.063 mm (EN-933-1) and filter (sieve) 0.19 mm—mass of dust passing through the 0.19 mm filter (sieve);
  ◦ Fine/rubber granule grading using filter (sieve) 0.19 mm—mass of material retained.
- Evaluation of chemical composition of non-soluble components with the use of EDX spectroscopy (Quanta Feg 250 SEM and EDS spectrum).

Due to the type of material and fine grain size of the filler, sieving was performed for a sealant diluted with a solvent using only a standard sieve of 0.063 and a filter (sieve) of 0.19 mm.

## 3. Results

### 3.1. Solvent Extraction of Joint Sealants

**Figure 2:**
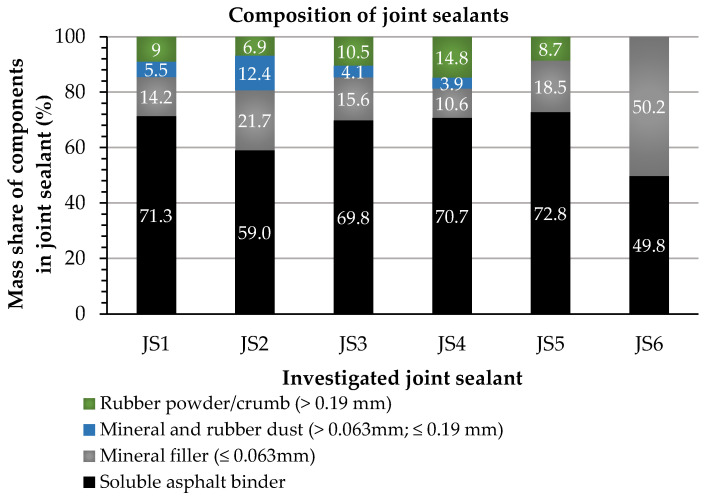
Composition (by mass) of components in investigated joint sealants after solvent extraction.

The composition of the investigated joint sealants (JS1–JS6) was determined on the basis of extractions performed with the use of tetrachloroethylene as a solvent (EN 12697-1, EN 12697-3+A1). In addition to their main component, which was the asphalt binder, mineral filler and rubber particles were found. The solvent extraction results are illustrated in Figure 2.

The current standard EN 14188-1 does not impose the percentages of the components of hot-applied joint sealants. The investigated joint sealants are therefore characterized by the highly varied content of the asphalt binder and other components (Figure 2).

The content of soluble asphalt binder in the investigated joint sealants ranged from 49.8 to 72.8%. In the sealants JS1, JS3, JS4, and JS5, it was close to 70%. In five joint sealants (JS1–JS5), the occurrence of rubber particles with a grain size above 0.19 mm with different content percentages (6.9–14.8%) was found. The identified crumb rubber contents were similar to those investigated by Gnatenko et al. [18] and Gong et al. [17] (5, 10, 15, and 20%) in the studies of asphalt-based sealants. The positive effects of the use of rubber crumb in joint sealants were also indicated in [25].

In the JS1–JS4 sealants, combined mineral and rubber dust particles (>0.063 mm; ≤0.19 m), which were difficult to separate into mineral and rubber parts, were also screened (3.9–12.4%).

The content of mineral filler passing the 0.063 mm sieve in sealants JS1–JS5 ranged from 10.6 to 21.7%. The JS6 joint sealant consisted of an asphalt binder and a mineral filler that passed through the 0.063 mm sieve (50.2/49.8%).

**Figure 3:**
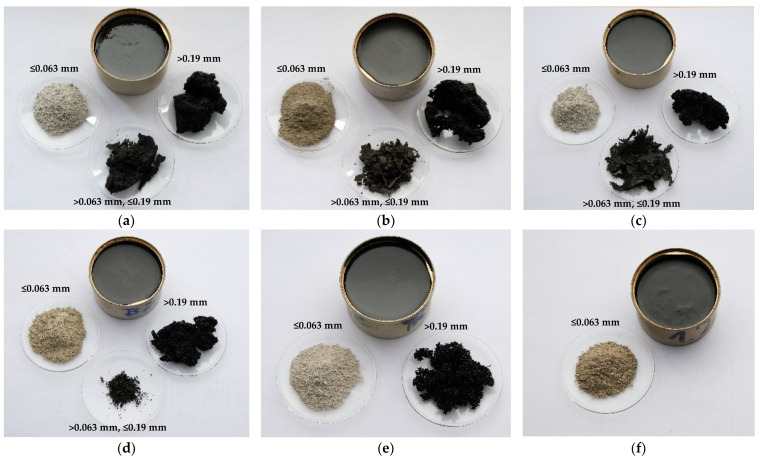
Photographs of component materials after extraction of joint sealants: (**a**) JS1; (**b**) JS2; (**c**) JS3; (**d**) JS4; (**e**) JS5; (**f**) JS6.

Figure 3 shows photographs of the separated component materials after the solvent extraction of the investigated hot-applied joint sealants.

A visual evaluation of the components separated from the joint sealants (Figure 3) indicated that mineral materials and rubber crumbs of various origins were used in their production, which may have affected the properties of the product. Also, the perceptible features of asphalt binder, i.e., the surface tension and shine, indicated its possible modification. In a further part of the paper, the chemical composition of the extracted components of the investigated sealants and their basic properties are analyzed.

### 3.2. Chemical Composition of Non-Soluble Components with the Use of EDX Spectroscopy

#### 3.2.1. Mineral Fillers

**Figure 4:**
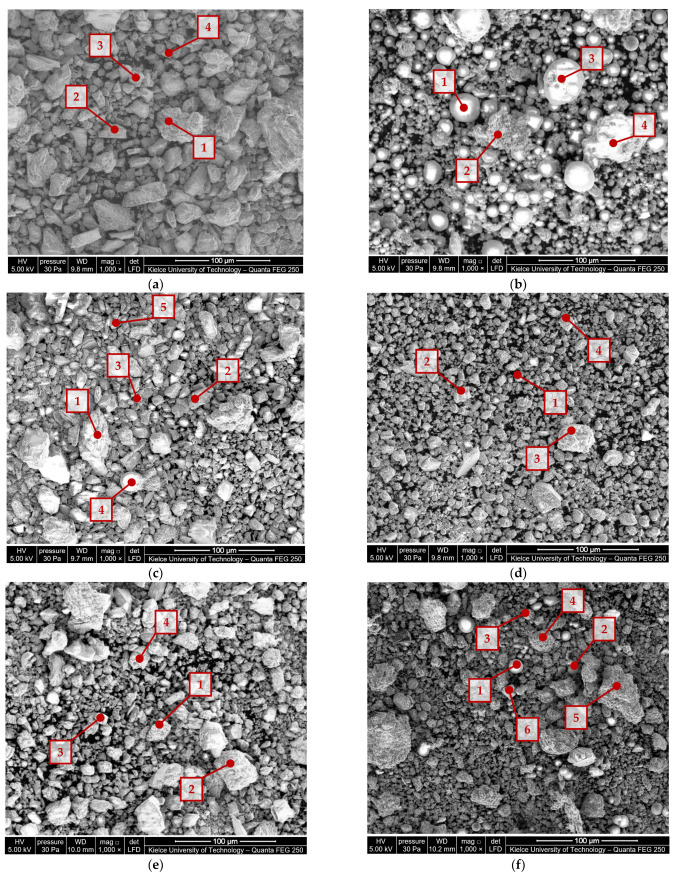
Scanning electron microscope images of the mineral fillers (≤0.063 mm) from joint sealants using a Quanta Feg 250 SEM and EDS spectrum at selected measuring points: (**a**) JS1; (**b**) JS2;(**c**) JS3; (**d**) JS4; (**e**) JS5; (**f**) JS6.

In order to check the homogeneity of the investigated material, a minimum of four measurement points were selected in the places of the occurrence of fragments differing in size, shape, or color in the macroscopic assessment. EDX (energy-dispersive X-ray) spectroscopy was used to validate the composition of the mineral fillers. The scanning electron microscope images of the mineral fillers extracted from the joint sealants are shown in Figure 4.

The chemical composition of the mineral fillers extracted from sealants JS1–JS6 is given in Table 3.

The analysis of the chemical composition (Figure 4, Table 3) showed the presence of calcium and silicon, as well as other elements of mineral origin in small amounts (<1.0%), such as sodium, magnesium, potassium, and other chemical compounds included in the composition. Traces of compounds from the insoluble asphalt binder and other components of the joint sealants were also detected.

In the mineral fillers derived from the joint sealants JS1, JS4, and JS5, the dominant share of calcium was found at all the measuring points, indicating a use of limestone filler. In the material obtained from the J3 and JS6 sealants, a similar chemical composition was identified in some of the measuring points (for the JS3 sealant, at points 1–3, and for the JS6 sealant, at points 3–6). For the JS2 joint sealant and other measuring points from the JS3 and JS6 sealants, silicon had the highest percentage in the investigated material, which indicates the use of mineral fillers of other origins in the production of joint sealants.

#### 3.2.2. Rubber Powder/Crumb

**Figure 5:**
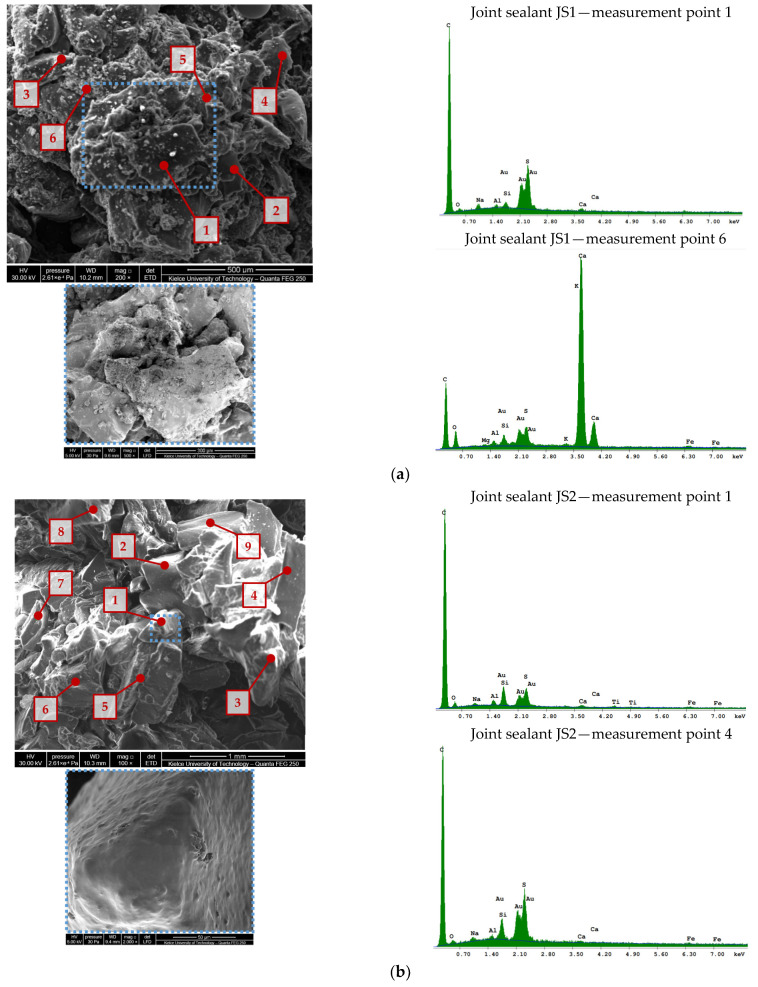
Scanning electron microscope image of rubber powder/crumb (>0.19 mm) from JS1 and JS2 joint sealants using a Quanta Feg 250 SEM and EDS spectrum at selected measuring points: (**a**) JS1; (**b**) JS2.

**Figure 6:**
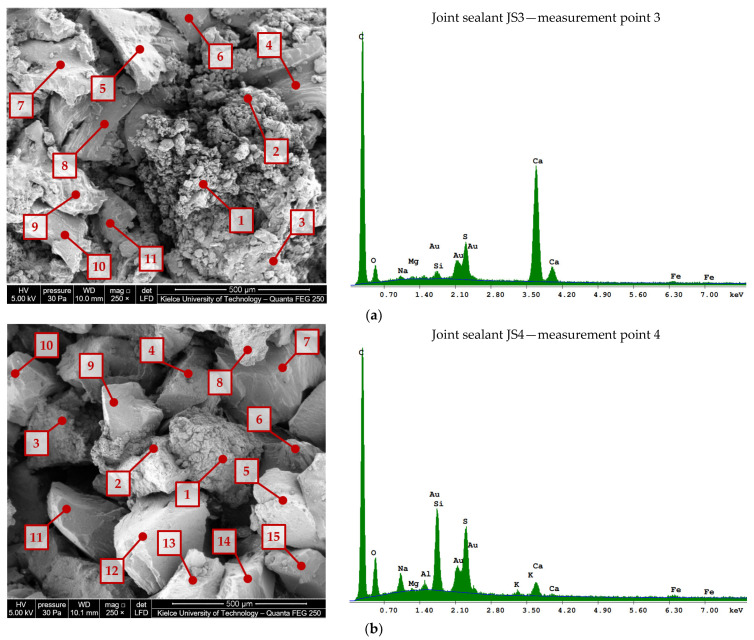
Scanning electron microscope image of rubber powder/crumb (>0.19 mm) from JS3 and JS4 joint sealants using a Quanta Feg 250 SEM and EDS spectrum at selected measuring points: (**a**) JS3; (**b**) JS4.

**Figure 7:**
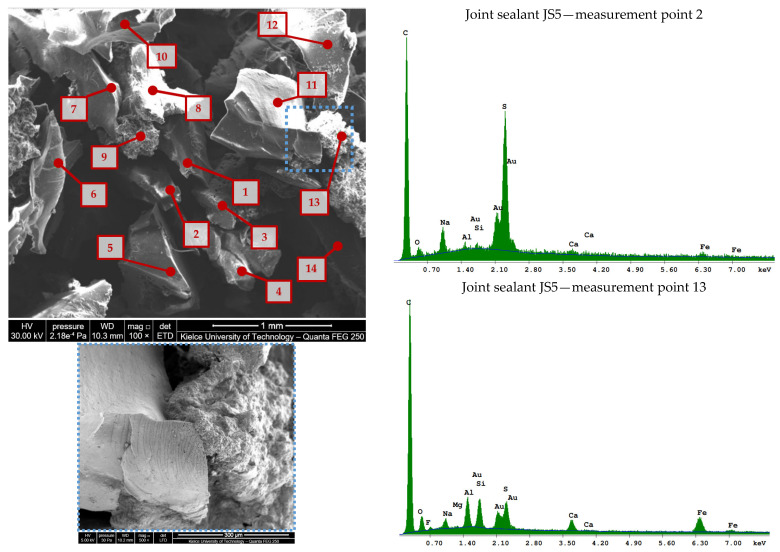
Scanning electron microscope image of fines/rubber granules (>0.19 mm) from JS5 joint sealants using the Quanta Feg 250 SEM and EDS spectrum at selected measuring points.

The scanning electron microscope images of the rubber powder/crumb extracted fromthe JS1–JS2 sealants are shown in Figure 5, those from JS3–JS4 are shown in Figure 6, and those from JS5 are shown in Figure 7.

The chemical composition of the fines/rubber granules extracted from sealants JS1–JS5 is given in Table 4 and Table 5.

In rubber from used car tires, there can be many components, such as natural and synthetic rubber, aromatic oils, fillers, sulfur, and other chemical additives [26]. The variation in the content of the rubber waste components, as well as the method of producing crumb rubber (cryogenic and mechanical method), significantly affects the physicochemical properties of rubber–asphalt binders [27]. Mechanically produced rubber particles have a large surface area and interact better with asphalt compared to rubber obtained using a cryogenic method [26].

Rubber waste can be characterized by various components and chemical additives. In order to identify the degree of variation in their composition and grinding method (cryogenic technology and mechanical grinding at an ambient temperature), 6 to 15 measurement points were selected. They were designated in the places of the occurrence of fragments differing in size, shape, or color in the macroscopic assessment. Selected fragments of the rubber particles are shown at ×500 (JS1, JS5) or ×2.000 (JS2) magnification.

The rubber particles identified in the JS2 joint sealant (Figure 5b) have a regular shape with smooth and glossy surfaces, indicating the possibility of the use of cryogenic technology to granulate the waste rubber ([28,29]). In other materials (JS1 and JS3–JS5), the appearance of the porous surface and the particle size indicate that a mechanical process (e.g., Figure 6b) or mixed methods (e.g., Figure 5a) were used. It is known that in order to obtain a very fine granular size, a combination of both cryogenic and mechanical processes must be used [30].

Due to the difficulties in fully separating mineral fillers from rubber, elements of mineral origin might have also appeared in the investigated material. The presence of elements of mineral origin, such as calcium, magnesium, and silicon, was found (Table 4 and Table 5). In addition to the components of hydrocarbon rubber, sulfur with a different content (1.19–10%) was also identified, which indicates the production of rubber crumbs from materials with different degrees of hardness.

### 3.3. Basic Properties of the Investigated Joint Sealants and Recovered Asphalt Binder

The evaluations of the penetration at 25 °C, the softening point, the Fraass breaking point, and the elastic recovery were performed to measure the effects of different types and compositions of joint sealants on their and the asphalt binders’ classical properties. Table 6 shows the test results of the joint sealants, and Table 7 presents the results obtained for the extracted asphalt binders. The cone penetration test at 25 °C was conducted only for the original joint sealants.

The determined values of the softening point (Table 6) correspond with the standard requirements [5] for hot-applied joint sealants (≥85 °C). For joint sealants JS1, JS2, JS3, and JS6, the results are also within the limits declared by their manufacturers (Table 1). In the case of the JS4 sealant, a value approx. 2 °C higher than that declared by the manufacturer was obtained. The JS5 sealant manufacturer did not indicate the declared values for this parameter.

A comparison with the standard requirements [5] for cone penetration shows that for one sealant, JS2, they were not met (40 to 100 × 0.1 mm). The obtained average value of this parameter was 25.6 °C, which indicates an increased hardness of this material. In the case of needle penetration, this parameter was also the lowest among all the tested sealants. It was also not included in the scope declared by the manufacturer (Table 1). In the case of the JS4 sealant, a value approx. 14 × 0.1 mm lower than that declared by the manufacturer was obtained.

The highest value of the Fraass breaking point characterized the JS1 and JS4 sealants. For the other materials, this parameter was lower than −25 °C. The Fraass breaking point test is not required according to EN 14188-1, but it is important for the assessment of the elasticity of the tested joint sealant at a low temperature.

The analysis of the results in Table 7 shows that the asphalt binders extracted from the joint sealants did not relate clearly to the types provided in the national annexes (PL) of standards EN 12591 (specifications for paving-grade bitumens) and EN 14023 (specification framework for polymer-modified bitumens), which are used for paving asphalt binders. This result may have been affected by the influence of the modification of the varying content of crumb rubber.

The results of the elastic recovery test at 25 °C in the case of sealants JS1 and JS3–JS6 indicated a very high rate of elastomeric modification similar to that seen in the highly modified bitumens produced in Poland according to the national annex of the PN-EN 14,023 standard; these are characterized by a high content of SBS polymer in an amount equaling about 7–7.5% m/m. The high elasticity of the binder ensures the proper operation of the elastic joint sealants, allowing for the transmission of the significant deformations and strains caused by both vehicle traffic and climatic factors. The JS2 sample failed before the end of the test but showed an almost immediate return to its original shape.

**Figure 8:**
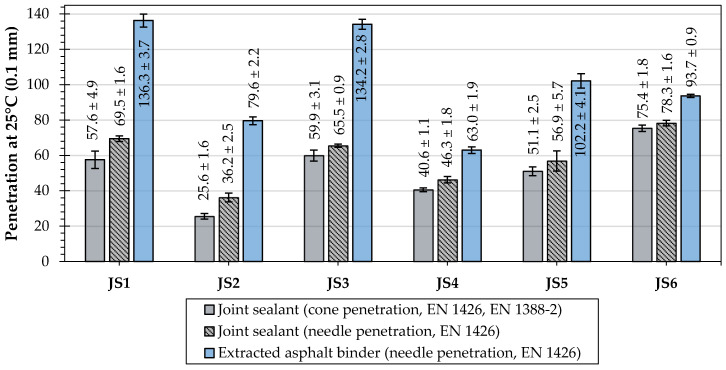
Results of penetration at 25 °C of the investigated joint sealants and extracted asphalt binders.

**Figure 9:**
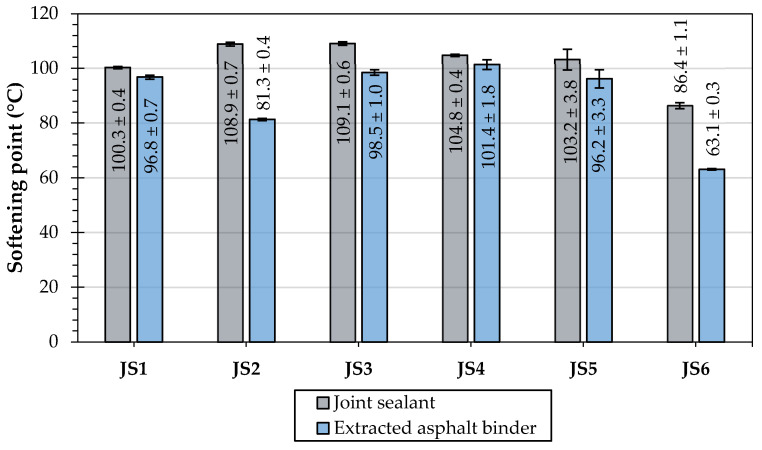
Results of softening point of the investigated joint sealant and soluble asphalt binders.

**Figure 10:**
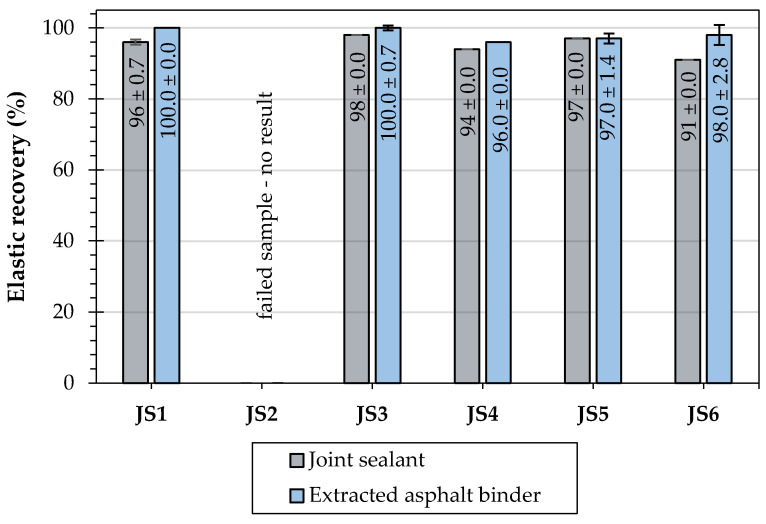
Results of elastic recovery of the investigated joint sealant and soluble asphalt binders.

Figure 8, Figure 9 and Figure 10 illustrate the mean values with the standard deviation (SD) of penetration, softening point, and elastic recovery of the tested joint sealants and extracted asphalt binders.

The cone penetration tests indicated significant variability in the consistency of the joint sealants (25.6 to 75.4 × 0.1 mm). This was also evident in the scope of the needle penetration and the diverse properties of the asphalt (63 to 136.3 × 0.1 mm). The softest asphalt binders were used for the N1 type joint sealants (JS1, JS3), but this did not significantly affect the properties of the joint sealants. The properties of the asphalt binders were, to varying degrees, transferred to the characteristics of the sealants (Figure 8).

The penetration and softening point tests indicated that the JS2 sealant was the hardest in consistency, while JS6 was the most susceptible to high temperatures. In addition, the JS2 sealant did not meet the requirements of the standard [5] nor the manufacturers’ declarations regarding cone penetration (40 to 100 × 0.1 mm). Such characteristics of the investigated sealant may indicate its reduced resistance to cracking.

The lowest softening point was determined for the JS6 joint sealant, which uses an asphalt binder with similarly low characteristics. At the same time, this sealant comprised the largest mass share of mineral filler in its composition (approx. 50%).

Both the joint sealants and the extracted bitumens had high average elastic recovery values that ranged from 91 to 98% for the joint sealants and 96 to 100% for the binders.

### 3.4. Evaluation of the FTIR Spectra of the Joint Sealants and Recovered Asphalt Binders

**Figure 11:**
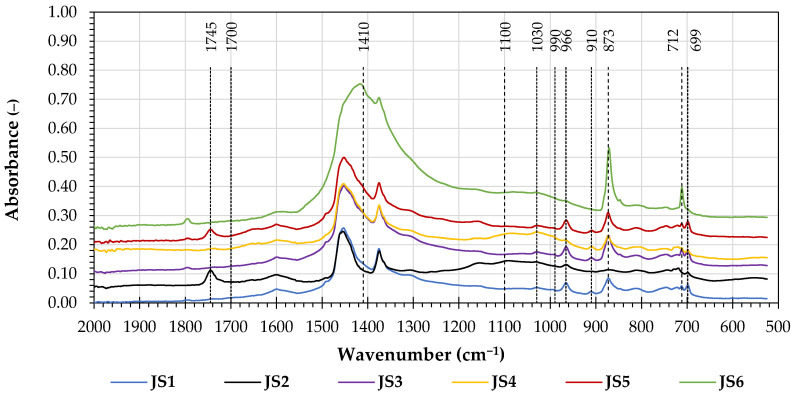
FTIR spectra of the investigated joint sealants.

The FTIR spectra of the evaluated joint sealants and the extracted soluble contents of the asphalt binders are presented in Figure 11.

Figure 11 presents the 2000 cm^−1^ to 500 cm^−1^ absorption bands of the overlayed spectra of the joint sealants.

The infrared spectra of the investigated joint sealants varied significantly and significantly differed from the typical FTIR spectra of bitumen. These differences were most likely due to the presence of fine mineral aggregates and rubber particles and possibly to other components of the joint sealants. In the infrared spectra of joint sealants JS1, JS3, JS4, JS5, and JS6, prominent absorption peaks in the 873 cm^−1^ and 712 cm^−1^ wavenumber regions were observed, accompanied by the rise of the absorption in the 1500–1300 cm^−1^ wavenumber region. These absorption bands could be attributed to the presence of calcium carbonate (presumably in the form of calcite), which is characterized by a strong and broad 1410 cm^−1^ peak (asymmetric C-O stretching) and two smaller peaks at 873 cm^−1^ (out-of-plane vibration) and 712 cm^−1^ (in-plane vibration) [31,32]. The JS2 and JS4 joint sealants clearly exhibited increased absorptions in the 1100 cm^−1^ and 500 cm^−1^ wavenumber regions, which were possibly associated with the presence of SiO_2_ [33,34].

These observations are mostly in line with the results of the EDS elemental analysis (shown in Table 3), which corroborates the significant presence of calcium in the fine residues from the joint sealants JS1, JS3, JS4, JS5, and JS6, as well as the silicon in the JS2 joint sealant. In this analysis it can be seen that the presence of SiO_2_ could not be easily established based on the FTIR spectra of the JS3 and JS6 sealants; this is possibly due to the effects of other adjacent absorbance bands (e.g., the strong and wide 1500-1300 cm^−1^ band related to the presence of CaCO_3_). On the other hand, the presence of SiO_2_ was not confirmed in the EDS analysis of the JS4 sealant; this could potentially be attributed to the point-like nature of the employed method of measurement. The summary and comparison of the chemical analyses performed using the FTIR and EDS methods are provided in Table 8.

**Figure 12:**
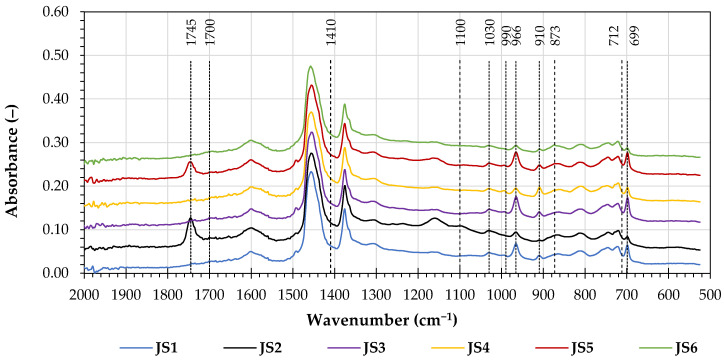
FTIR spectra of the asphalt binders extracted from the investigated joint sealants.

Figure 12 presents the 2000 cm^−1^ to 500 cm^−1^ absorption bands in the FTIR spectra of the soluble asphalt binders extracted from the joint sealants.

All the FTIR spectra of the extracted asphalt binders show low, comparable peak heights in the 1700 cm^−1^ and 1030 cm^−1^ wavenumber regions associated with carbonyl and sulfoxide compounds, indicating that they were not exposed to a significant amount of oxidation.

All of the extracted binders exhibited significant peaks at 966 cm^−1^, indicating the presence of butadiene structures; at 990 cm^−1^ and 910 cm^−1^, which are characteristic of vinyl groups; and at 699 cm^−1^, which is characteristic of styrene structures. These responses indicate the presence of styrene–butadiene rubber in the samples of the tested soluble material. Additionally, the asphalt binder extracted from the JS2 and JS5 joint sealants registered prominent peaks at the 1745 cm^−1^ wavenumber, which is typically seen in bio-oil derivatives (e.g., fatty acid methyl esters) and may correspond to the stretching of the -C=O in ester functional groups.

## 4. Conclusions

The present study considered the chemical composition and basic performance properties of hot-applied joint sealants and their components. Based on the study, the conclusions were as follows:

- The solvent extraction showed that the investigated joint sealants were characterized by different contents of soluble asphalt binder (49.8 to 72.8%), mineral filler that passed through a 0.063 mm sieve (10.6 to 50.2%), and other components, e.g., crumb rubber.
- The use of the EDX spectroscopy method indicated significantly varied chemical composition and the origin of the non-soluble components (mineral fillers and rubber crumbs) used in the production of the joint sealants.
- The tests showed similar properties of the sealants in terms of elastic recovery at 25 °C (91 to 98%), despite the different contents and chemical compositions of their individual components, the results indicated a very high degree of elastomeric modification of the bitumens used in the sealants and/or the effect of rubber modification, regardless of its type (normal or elastic).
- The determined values of the softening point corresponded with the European standard requirements for all investigated hot-applied joint sealants (≥85 °C). The lowest softening points were determined for the joint sealant that used asphalt binders with similarly low characteristics and had the largest mass share of mineral filler in its composition (approx. 50%).
- The cone penetration tests indicated significant variability in the consistency of the joint sealants (25.6 to 75.4 × 0.1 mm). One of the sealants met neither the requirements of the applicable European standard nor the manufacturer’s declaration for cone penetration (40 to 100 × 0.1 mm).
- The FTIR spectra of the extracted asphalt binders showed significant peaks at 966 cm^−1^, indicating the presence of butadiene structures; at 990 cm^−1^ and 910 cm^−1^, which are characteristic of vinyl groups; and at 699 cm^−1^, which is characteristic of styrene structures.
- The use of EDX spectroscopy and Fourier transform infrared spectroscopy (FTIR) methods allowed us to perform analyses of the chemical composition of the joint sealants and their individual soluble and non-soluble components for comparative purposes, as well as enabling the determination of their optimal composition.
- The FTIR method was shown to be able to indicate the presence of calcareous fillers in asphaltic materials; the spectral response of siliceous aggregates was too weak to reliably confirm their presence in the joint sealants, particularly when fine calcareous aggregates were also present.
- It is possible to regulate the selected properties of the joint sealant by changing the chemical composition and the proportion of its components.
- Joint sealants are complex formulations and, given a broad range of base asphalt binders, modifiers, fillers, and other non-soluble additives, their composition may vary widely, while still fulfilling the performance specifications.

In the present study, the composition of different crack sealing materials was inferred and juxtaposed with their performance. The investigation utilized an expanded scope of tests and identification methods to analyze the joint sealants, including material extraction and the testing of the properties of the component materials. In the investigation, it was found that EDS and FTIR together with the solvent extraction, and separation provided insights into the composition of the joint sealants and the types of mineral materials used. It was also shown that radically different compositions of the crack sealants could result in a comparatively similar engineered final performance of the tested material.

The combined use of these analysis methods could be used in, for example, forensic investigations to evaluate the fine aggregate composition used in asphaltic materials, such as asphalt mixtures and joint sealants. The results will provide a basis for subsequent research to develop an experiment plan to evaluate the effects of the composition of different joint sealants on their functional and durability characteristics, including economic and sustainability considerations.

## Figures and Tables

**Table 1:** Selected required characteristics of joint sealant according to EN 14188-1 [5] and declarations by manufacturers.

Material Properties	EN 14188-1 [5] Requirements		Characteristics of Tested Joint Sealant and Declaration by Manufacturers						Testing Method
			JS1	JS2	JS3	JS4	JS5	JS6	
Type of hot-applied joint sealant	N1	N2	N1	N2	N1	N2	N2	N2	[5]
Density at +25 °C, in Mg/m^3^	manufacturer’s declaration		1.15 ± 0.05	1.12	1.15 ± 0.05	1.2	no dec.	1.2 ± 0.1	EN 13880-1
Softening point, ring, and ball, in °C	≥85	≥85	98 ± 8	≥ 85	98 ± 8	102	no dec.	≥85	EN 1427
Cone penetrationat 25 °C, in 0.1 mm	40to 130	40to 100	60 ± 10	40to 100	60 ± 10	54	40 to 100	40to 100	EN 13880-2
Penetration and recovery (resilience) at +25 °C, in %	≥60	≤60	65 ± 5	<60	65 ± 5	59	≤60	≤60	EN 13880-3
Heat stability/change in penetration value at +70 °C/168 h									EN 13880-4
Cone penetration, in 0.1 mm	40 to 130	40 to 100	<70	no dec.	<70	no dec.	40 to 100	40 to 100	
Penetration and recovery (resilience), in %	≥60	≤60	>60	no dec.	>60	no dec.	≤60	≤60	
Flow resistance, and initial and heat degradation at +60 °C, 5 h, 75° angle, in mm	≤2	≤3	<2	<3	<2	≤3	≤3	≤3	EN 13880-5
Compatibility with asphalt pavements at +60 °C, 72 h	No failures in adhesion and no formation of any oily exudate								EN 13880-9
Bonding strength									EN 13880-13
Total extension within 5 h, in mm	≥5	≥5	≥5	no dec.	≥5	5	≥5	≥5	
Test temperature, in °C	−25	−20	−20	no dec.	−20	−20	−20	−20	
Maximum tension, in N/mm^2^	1.00	0.75	<0.6	no dec.	<0.6	0.34	non-failure	0.75	
Final tension, in N/mm^2^	≤0.15	not req.	≤0.15	not req.	≤0.15	not req.	not req.	not req.	
Cohesion									EN 13880-10
Extension, in mm	18	18	18	no dec.	18	no dec.	18	18	
Extension, in %	75	75	75	no dec.	75	no dec.	75	75	
Number of cycles	3	3	3	no dec.	3	no dec.	3	3	
Test temperature, in °C	−20	0	0	no dec.	0	no dec.	0	0	
Maximum tension, in N/mm^2^	0.48± 0.1	0.48± 0.1	0.48 ± 0.1	no dec.	0.48± 0.1	no dec.	non-failure	0.48± 0.1	
Legend:	no dec.	—	No declaration by manufacturer;						
	not req.	—	No required acc. to EN 14188-1 [5].						

**Table 2:** Distillation condition used in the testing methodologies for recovery of soluble bitumen.

Solvent		First Phase		Second Phase		Extra Temperature (°C)
Description	Boiling Point (°C)	Temperature (°C)	Pressure (kPa)	Temperature (°C)	Pressure (kPa)	
Tetrachloroethylene	121.0	110	40	160	2.0	180

**Table 3:** Mass share of elements detected in EDS analysis of mineral fillers (≤0.063 mm) extracted from joint sealants at measuring points.

Joint Sealant	Measuring Point	Mass Share of Elements in the Investigated Material Wt (%)													
		C	O	Al	Na	Si	S	Ca	Mg	K	Ti	Fe	P	Mn	Total
JS1	1	9.53	41.04	0.80	-	-	-	48.63	-	-	-	-	-	-	100
	2	10.60	43.68	0.70	-	-	-	45.01	-	-	-	-	-	-	100
	3	12.74	49.16	0.67	-	0.34	-	37.09	-	-	-	-	-	-	100
	4	11.58	47.18	0.31	-	-	-	40.92	-	-	-	-	-	-	100
JS2	1	-	39.78	16.56	0.51	34.07	-	1.70	1.21	2.01	1.48	2.69	-	-	100
	2	3.08	43.17	19.76	0.65	28.25	-	0.87	0.62	1.70	-	1.90	-	-	100
	3	-	37.36	25.31	0.46	30.83	-	0.53	0.45	1.99	1.48	1.58	-	-	100
	4	2.26	42.61	21.17	0.21	27.24	-	0.48	0.44	1.28	3.08	1.22	-	-	100
JS3	1	9.55	39.97	0.73	-	0.70	-	48.50	0.56	-	-	-	-	-	100
	2	10.59	43.97	-	-	-	-	45.44	-	-	-	-	-	-	100
	3	11.35	45.12	0.53	-	-	-	42.17	0.83	-	-	-	-	-	100
	4	2.92	40.88	19.53	0.16	28.30	-	3.11	0.97	1.20	0.66	2.26	-	-	100
	5	8.46	42.25	10.25	1.02	26.02	-	7.26	1.05	2.17	-	1.52	-	-	100
JS4	1	12.76	48.66	2.26	-	3.89	-	28.97	1.97	0.53	-	0.96	-	-	100
	2	12.19	42.76	2.10	-	3.09	-	36.19	2.40	0.52	-	0.76	-	-	100
	3	9.43	40.64	0.95	-	0.80	-	45.08	0.93	-	-	2.17	-	-	100
	4	7.13	43.14	4.98	-	7.22	-	31.12	3.86	1.07	-	1.48	-	-	100
JS5	1	8.91	38.95	0.96	-	0.99	-	49.44	0.76	-	-	-	-	-	100
	2	9.84	44.74	0.57	-	-	-	43.89	0.95	-	-	-	-	-	100
	3	13.61	51.54	0.60	-	0.35	-	33.31	0.59	-	-	-	-	-	100
	4	9.44	41.71	0.49	-	-	-	48.37	-	-	-	-	-	-	100
JS6	1	3.81	35.04	17.00	0.71	22.33	-	9.73	3.84	0.78	0.88	5.87	-	-	100
	2	4.51	37.17	18.69	1.29	22.43	0.72	4.63	1.51	2.33	0.60	6.12	-	-	100
	3	45.64	22.37	4.47	0.36	6.94	0.46	14.12	0.52	0.1	-	1.23	3.79	-	100
	4	9.35	39.08	0.98	-	0.72	-	48.98	0.89	-	-	-	-	-	100
	5	11.48	46.25	0.87	-	0.87	0.18	39.54	0.80	-	-	-	-	-	100
	6	4.66	30.92	2.06	-	1.53	2.52	34.30	18.89	-	-	3.64	-	1.47	100
Legend:			- The highest percentage of the element in the investigated material;												
			- Second percentage of element in the test material;												
			- The third and subsequent percentage of the element in the investigated material (≥1.0%);												
			- The third and subsequent percentage of the element in the investigated material (<1.0%);												
		-	- Lack of element in the investigated material.												

**Table 4:** Mass share of elements detected in EDS analysis of fines/rubber granules (>0.19 mm) from joint sealants JS1—JS3 at measuring points.

Joint Sealant	Measuring Point	Mass Share of Elements in the Investigated Material Wt (%)													Total
		C	O	Al	Na	Si	S	Ca	Mg	K	Ti	Fe	F	Au	
JS1	1	81.23	2.26	0.31	0.90	0.52	5.46	0.40	-	-	-	-	-	8.92	100
	2	78.53	4.50	0.31	0.81	5.90	3.94	0.31	-	-	-	-	-	5.68	100
	3	80.05	3.37	0.21	0.65	0.22	5.83	0.80	-	-	-	-	-	8.87	100
	4	77.00	5.79	0.25	0.79	0.53	4.93	2.90	-	-	-	0.56	-	7.25	100
	5	80.65	3.15	-	0.81	2.06	5.29	0.49	-	-	-	-	-	7.55	100
	6	34.85	15.95	0.84	-	1.42	2.85	35.12	0.41	0.53	-	0.75	-	7.28	100
JS2	1	84.50	4.27	0.64	0.52	2.21	2.39	0.32	-	-	0.31	0.40	-	4.45	100
	2	81.81	4.51	0.31	0.54	4.84	2.98	0.55	-	-	-	-	-	4.47	100
	3	48.65	15.79	3.09	0.45	3.73	1.82	18.30	0.33	-	-	1.39	-	6.45	100
	4	79.60	2.57	0.36	0.61	1.82	5.27	0.26	-	-	-	0.28	-	9.24	100
	5	67.14	5.76	4.31	0.33	8.14	1.85	2.18	0.39	0.62	0.33	1.61	-	7.32	100
	6	88.15	3.61	-	0.61	0.88	2.39	-	-	-	-	-	-	4.37	100
	7	69.74	8.86	0.96	0.60	3.92	2.81	7.15	0.41	0.24	-	0.85	-	4.46	100
	8	89.66	3.76	0.15	0.46	0.25	2.25	-	-	-	-	-	-	3.47	100
	9	78.81	6.36	1.38	0.41	4.26	2.20	0.32	0.07	0.31	-	0.59	-	5.28	100
JS3	1	45.55	18.71	1.80	0.81	2.58	2.82	19.56	0.40	0.66	-	0.89	0.82	5.38	100
	2	24.14	16.01	0.75	-	1.90	3.74	45.72	-	0.83	-	-	-	6.93	100
	3	67.68	9.65	-	0.52	0.59	3.08	12.88	0.21	-	-	0.41	-	4.98	100
	4	83.12	2.19	0.24	0.35	0.23	5.16	1.02	-	-	-	-	-	7.69	100
	5	75.72	7.85	-	0.89	5.84	4.57	0.68	-	-	-	-	-	4.45	100
	6	74.88	6.00	0.22	0.69	0.59	6.70	2.85	-	-	-	-	-	8.07	100
	7	85.53	2.47	-	1.15	-	4.71	0.46	-	-	-	-	-	5.68	100
	8	83.75	4.64	0.11	0.76	0.18	4.95	1.14	0.12	-	-	-	-	4.34	100
	9	84.73	4.14	-	0.92	-	3.94	0.98	-	-	-	-	-	5.28	100
	10	47.62	12.95	0.15	0.55	25.28	5.25	0.54	0.17	-	-	-	-	7.47	100
	11	50.63	7.47	0.48	0.63	25.46	6.07	0.79	0.23	-	-	-	-	8.25	100
Legend:			- The highest percentage of the element in the investigated material;												
			- Second percentage of element in the test material;												
			- The third and subsequent percentage of the element in the test material (≥1.0%);												
			- The third and subsequent percentage of the element in the test material (<1.0%);												
			- Signal from the gold coating on the sample;												
		-	- Lack of element in the test material.												

**Table 5:** Mass share of elements detected in EDS analysis of fines/rubber granules (>0.19 mm) from joint sealants JS4 and JS5 at measuring points.

Joint Sealant	Measuring Point	Mass Share of Elements in the Investigated Material Wt (%)												Total
		C	O	Al	Na	Si	S	Ca	Mg	K	Fe	F	Au	
JS4	1	48.93	16.13	1.26	0.77	6.91	2.95	14.87	1.41	0.60	1.23	-	4.94	100
	2	52.65	17.51	0.73	1.05	3.96	3.29	11.58	0.46	0.24	0.71	-	7.84	100
	3	11.62	32.16	1.29	-	1.51	-	47.43	1.24	0.35	1.16	-	3.23	100
	4	67.89	13.91	0.40	1.76	4.65	4.33	1.14	0.12	0.32	0.40	-	5.07	100
	5	72.28	8.69	0.21	2.57	2.63	4.95	1.58	0.23	0.22	0.32	-	6.31	100
	6	78.18	7.10	0.17	2.57	0.56	5.23	0.57	-	-	-	-	5.62	100
	7	82.03	5.49	0.33	1.19	0.31	5.01	0.40	-	0.27	-	-	4.97	100
	8	69.93	7.39	0.19	1.28	6.14	5.53	0.61	-	0.35	0.52	-	8.06	100
	9	50.05	12.67	0.18	2.21	19.64	7.26	-	-	-	-	-	7.98	100
	10	77.71	6.09	0.36	2.72	0.91	4.86	0.56	0.21	0.27	0.50	-	5.82	100
	11	73.07	4.00	0.25	3.08	4.89	8.26	0.45	-	0.58	-	-	5.42	100
	12	72.32	4.89	0.29	3.74	0.19	10.00	0.40	0.10	-	-	-	8.07	100
	13	76.71	9.01	0.19	1.03	0.53	2.95	2.63	0.20	0.31	-	-	6.44	100
	14	78.74	6.38	0.27	2.22	0.68	5.16	0.46	-	-	-	-	6.10	100
	15	72.17	5.66	0.43	3.04	0.66	8.32	0.82	0.39	0.30	0.49	-	7.71	100
JS5	1	79.23	4.61	0.16	2.54	0.27	5.32	0.40	0.16	-	0.42	-	6.89	100
	2	74.33	4.09	0.32	2.52	0.23	9.87	0.45	-	-	0.76	-	7.45	100
	3	75.60	9.12	0.35	1.07	0.51	2.25	2.56	0.18	-	2.18	1.68	4.51	100
	4	84.82	5.63	-	2.80	0.21	3.16	-	-	-	-	-	3.40	100
	5	74.44	9.56	0.19	2.22	0.36	2.71	0.13	-	-	1.48	1.71	7.20	100
	6	67.97	8.59	0.68	1.99	3.26	5.07	0.26	0.95	-	0.74	-	10.49	100
	7	57.09	19.82	0.32	2.05	10.39	2.43	0.28	0.09	-	0.98	1.02	5.53	100
	8	82.92	7.55	0.89	0.93	0.22	1.61	0.62	-	-	0.84	-	4.42	100
	9	86.15	6.08	0.64	0.29	-	1.19	0.46	-	-	0.60	-	4.59	100
	10	67.71	6.90	0.47	2.49	4.41	4.66	0.22	0.06	-	0.84	0.73	11.51	100
	11	85.12	5.61	0.77	1.22	0.22	1.95	-	-	-	0.60	-	4.51	100
	12	69.80	7.34	0.50	1.25	5.33	3.84	0.62	0.08	0.21	1.09	0.70	9.23	100
	13	73.18	7.88	2.58	1.31	2.39	2.38	1.19	0.15	-	3.56	0.75	4.62	100
	14	83.37	3.25	0.21	0.88	0.07	4.90	0.43	0.12	-	0.53	-	6.25	100
Legend:			- The highest percentage of the element in the investigated material;											
			- Second percentage of element in the test material;											
			- The third and subsequent percentage of the element in the test material (≥1.0%);											
			- The third and subsequent percentage of the element in the test material (<1.0%)											
			- Signal from the gold coating on the sample;											
		-	- Lack of element in the test material.											

**Table 6:** Test results of joint sealants.

Property	Unit of Measure	Valid N	Joint Sealant						Testing Method
			JS1	JS2	JS3	JS4	JS5	JS6	
			Mean ± SD						
Cone penetrationat 25 °C	0.1 mm	5	57.6 ± 4.9	25.6 ± 1.6	59.9 ± 3.1	40.6 ± 1.1	51.1 ± 2.5	75.4 ± 1.8	EN 1426 EN 1388-2
Needle penetration at 25 °C	0.1 mm	5	69.5 ± 1.6	36.2 ± 2.5	65.5 ± 0.9	46.3 ± 1.8	56.9 ± 5.7	78.3 ± 1.6	EN 1426
Softening point, ring, and ball	°C	4	100.3 ± 0.4	108.9 ± 0.7	109.1 ± 0.6	104.8 ± 0.4	103.2 ± 3.8	86.4 ± 1.1	EN 1427
Fraass breaking point	°C	3	<−25.0	−24.3 ± 0.6	<−25.0	−21.3 ± 1.5	<−25.0	<−25.0	EN 12593
Elastic recovery at 25 °C	%	2	96.0 ± 0.7	62.0 mm; 48.0 mm *	98.0 ± 0.0	94.0 ± 0.0	97.0 ± 0.0	91.0 ± 0.0	EN 13398

* The sample failed before the end of test.

**Table 7:** Test results of asphalt binders extracted from investigated joint sealants.

Property	Unit of Measure	Valid N	Soluble Asphalt Binder from Joint Sealant						Testing Method
			JS1	JS2	JS3	JS4	JS5	JS6	
			Mean ± SD						
Needle penetration at 25 °C	0.1 mm	5	136.3 ± 3.7	79.6 ± 2.2	134.2 ± 2.8	63.0 ± 1.9	102.2 ± 4.1	93.7 ± 0.9	EN 1426
Softening point, ring, and ball	°C	4	96.8 ± 0.7	81.3 ± 0.4	98.5 ± 1.0	101.4 ± 1.8	96.2 ± 3.3	63.1 ± 0.3	EN 1427
Fraass breaking point	°C	3	−24.7 ± 0.6	<−25.0	<−25.0	−24.7 ± 0.6	<−25.0	<−25.0	EN 12593
Elastic recovery at 25 °C	%	2	100.0 ± 0.0	132.8 mm; 178.2 mm *	100.0 ± 0.7	96.0 ± 0.0	97.0 ± 1.4	98.0 ± 2.8	EN 13398

* The sample failed before the end of test.

**Table 8:** Inferred chemical composition of fillers present in the investigated joint sealants.

Joint Sealant	Calcareous Fillers (e.g., CaCO_3_)		Siliceous Fillers (e.g., SiO_2_)	
	FTIR	EDS	FTIR	EDS
JS1	x	x		
JS2			x	x
JS3	x	x		x
JS4	x	x	x	
JS5	x	x		
JS6	x	x		x

## Data Availability

Data available upon request.
